# First use of Trumenba (MenB-fHbp) vaccine to control a nursery outbreak of serogroup B invasive meningococcal disease involving children previously immunised with Bexsero (4CMenB), England, November 2023

**DOI:** 10.2807/1560-7917.ES.2026.31.3.2500431

**Published:** 2026-01-22

**Authors:** Kirsty Foster, Emma J Heymer, Helen Campbell, Emma Wilson, Jess Baldasera, Jay Lucidarme, Stephen A Clark, Xilian Bai, Shazaad Ahmad, Ray Borrow, Shamez N Ladhani

**Affiliations:** 1 North East Health Protection Team, UK Health Security Agency, Newcastle upon Tyne, United Kingdom; 2 Immunisation and Vaccine Preventable Diseases Division, UK Health Security Agency, Colindale, London, United Kingdom; 3 North West Health Protection Team, UK Health Security Agency, Preston, United Kingdom; 4 Meningococcal Reference Unit, UK Health Security Agency, Manchester Royal Infirmary, Manchester, United Kingdom; 5 Virology Department, Manchester Medical Microbiology Partnership, Manchester University NHS Foundation Trust, Manchester, United Kingdom; 6 Division of Evolution and Genomic Sciences, School of Biological Sciences, University of Manchester, Manchester, United Kingdom; 7 Centre for Neonatal and Paediatric Infections (CNPI), City St. George’s University of London, London, United Kingdom

**Keywords:** MenB, outbreak, MenB-fHbp, 4CMenB, vaccination, Nursery

## Abstract

In November 2023, the UK Health Security Agency was notified of PCR-confirmed group B (MenB) invasive meningococcal disease (IMD) in a 3-year-old child (Case A), followed by probable IMD in a 2-year-old (Case B, culture and PCR tests negative) attending the same nursery. An incident management team (IMT) was convened. Both children were fully vaccinated with the MenB vaccine 4CMenB (Bexsero, GSK Biologicals). All 39 children attending the nursery and nine staff received ciprofloxacin chemoprophylaxis preceded by pharyngeal swabbing. Pharyngeal swabbing yielded two MenB isolates matching Case A. Antibiotic sensitivity testing and assessment of 4CMenB vaccine coverage using the meningococcal antigen typing system (MATS) revealed the strain was not covered by the 4CMenB vaccine. Although the alternative MenB vaccine, MenB-fHbp (Trumenba, Pfizer), is only licensed from 10 years and has never been given to children previously immunised with 4CMenB, the IMT considered the benefits of outbreak control outweighed potential risks. Two doses were given 4 weeks apart to 38 children (one family declined) and all staff; there were no serious adverse events. Our findings highlight the utility of swabbing to identify outbreak strains and provide first evidence for safe use of the MenB-fHbp vaccine in children previously vaccinated with 4CMenB.

**Key public health message**

**What did you want to address in this study and why?**

We wanted to investigate the use of a meningococcal B (MenB) vaccine (MenB-fHbp, Trumenba) to control an invasive meningococcal disease (IMD) outbreak in a nursery caused by an MenB strain not covered by the 4CMenB (Bexsero) vaccine offered to infants in the UK national immunisation programme. MenB-fHbp, however, is not licensed for children under 10 years and has never been given to children previously immunised with 4CMenB.

**What have we learnt from this study?**

The MenB-fHbp vaccine was safely given to 38 children aged 1–9 years who had previously been immunised with 4CMenB in infancy. The nature, rates and duration of symptoms reported after MenB-fHbp vaccination were consistent with known side-effects reported previously for both vaccines.

**What are the implications of your findings for public health?**

In countries and regions with established childhood 4CMenB immunisation programmes, childhood IMD cases and outbreaks caused by MenB strains that are not covered by the 4CMenB vaccine are likely to increase. In such situations, supplementing antibiotic prophylaxis with MenB-fHbp vaccination could offer broader protection against IMD, even in children previously vaccinated with 4CMenB.

## Background

Invasive meningococcal disease (IMD) is rare but serious and typically manifests as meningitis, septicaemia or both [1]. The pathogen responsible for IMD, *Neisseria meningitidis*, is a Gram-negative diplococcus that is commonly carried in the nasopharynx of healthy individuals, especially teenagers who have the highest meningococcal nasopharyngeal carriage rates [1].

Twelve different meningococcal serogroups have been recognised based on their distinct polysaccharide capsule. Serogroups A, B, C, W, X and Y are responsible for nearly all IMD cases in different parts of the world [1]. In the United Kingdom (UK), serogroup B meningococci (MenB) are responsible for most IMD cases, especially in recent years, because of the excellent herd protection provided by the nationally-funded adolescent meningococcal conjugate ACWY vaccine programme [2].

The incidence of MenB IMD is highest in the first year of life and then declines in subsequent years, with a smaller second peak in teenagers and young adults [1]. In September 2015, the UK became the first country in the world to implement the 4CMenB vaccine (Bexsero, GSK Biologicals, Rixensart, Belgium), a four-component broad-spectrum protein-based MenB vaccine, into the national infant immunisation programme. The 4CMenB vaccine primarily targets meningococcal surface proteins. However, it does not protect against all MenB disease because some MenB strains may not possess any of the antigens included in the 4CMenB vaccine; also, there is wide antigenic and expression variability of these proteins on the meningococcal cell surface [3]. Prior to its implementation, the 4CMenB vaccine was predicted to protect against 73–88% of MenB strains. After 3 years of the national infant 4CMenB immunisation programme, there was a 75% reduction in MenB cases in children eligible for vaccination, from 253 cases among ca. 650,000 infants in 2015 to 63 cases in 2018 [3]. The 4CMenB vaccine is also the preferred vaccine for MenB outbreak control in the UK because it is licensed from 2 months of age, has proven effectiveness in the field and is more readily available due to its inclusion in the UK national immunisation programme.

Like many other respiratory pathogens, IMD incidence declined during the COVID-19 pandemic restrictions and then increased after these were lifted. Most of the increase was due to MenB [4], which was responsible for 88% (301/ 341) of IMD cases during the 2023/24 epidemiological year [5].

Most IMD cases are sporadic, but occasional clusters and outbreaks occur, usually in households, but also in educational settings such as schools, universities and military barracks. In the UK, there are published guidelines for the public health management of IMD cases, clusters and outbreaks, with specific recommendations for the use of antibiotic prophylaxis and meningococcal vaccines to prevent additional IMD cases [6]. Vaccine selection depends on the meningococcal serogroup responsible for the outbreak. For MenB strains, vaccine coverage can be predicted using phenotypic investigations such as the Meningococcal Antigen Typing System (MATS) or genotypic investigations such as the genetic (g) MATS / Meningococcal Deduced Vaccine Antigen Reactivity index (gMATS/MenDeVar index). If the strain is predicted to be not covered by the 4CMenB vaccine, another MenB vaccine, MenB-fHbp (Trumenba, Pfizer, New York, United States), may be used.

The MenB-fHbp vaccine is also a broad-spectrum protein-based vaccine, composed of two recombinant lipidated factor H-binding protein (fHbp) variants (subfamily A and B). It is estimated to provide protection against more than 90% of invasive MenB strains but has so far not been included in any national immunisation programme; real-world data on protection are, therefore, lacking [7,8]. Additionally, although a single clinical trial has assessed the immunogenicity of the MenB-fHbp vaccine in 1–9-year-olds [9], it is only licensed from 10 years of age. Importantly, too, there are no data on the use of the MenB-fHbp vaccine in individuals of any age who were previously vaccinated with the 4CMenB vaccine. The two protein-based meningococcal vaccines offer vaccinated individuals direct protection against IMD but are not effective in preventing carriage acquisition or clearing carriage and, thus, are unable to interrupt transmission or provide indirect (herd) protection for unvaccinated individuals [3,10].

## Outbreak detection

In November 2023, two invasive meningococcal disease (IMD) cases were identified in previously healthy children attending the same nursery in northern England within 4 days of each other. Case A was a 3-year-old who was hospitalised with fever, vomiting and a non-blanching rash, and was reported to the UK Health Security Agency (UKHSA) local health protection team (HPT) as a probable IMD case 4 days after symptom onset. The diagnosis was confirmed by a positive EDTA blood PCR for MenB. Three days later, a 2-year-old child from the same nursery (Case B) was hospitalised with fever and a non-blanching rash. Case B was notified to the HPT as a probable IMD case with subsequent cultures and meningococcal PCR tests negative for *N. meningitidis* (Figure and Supplementary Table S1). Both children had previously received three 4CMenB vaccine doses at 8 weeks, 16 weeks and 1 year as part of the national childhood immunisation programme.

**Figure:**
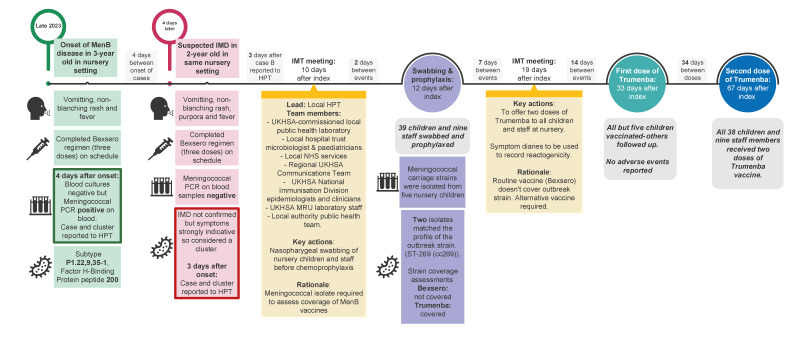
Timeline of events and public health actions following an invasive meningococcal disease outbreak in a nursery, England, November 2023 (n = 2 cases)

HPT: health protection team; IMD: invasive meningococcal disease; IMT: incident management team; MRU: Meningococcal Reference Unit; NHS: National Health Service; UKHSA: United Kingdom Health Security Agency.

Bexsero: 4CMenB vaccine (Bexsero, GSK Biologicals, London, UK).

Trumenba: MenB-fHbp vaccine (Trumenba, Pfizer, New York, United States).

Here we describe the public health response following this cluster of two IMD cases (one confirmed, one probable) in an English nursery, including antibiotic chemoprophylaxis, pharyngeal swabbing and the first use of the MenB-fHbp vaccine in young children previously vaccinated with 4CMenB. Given the potential for increased reactogenicity in previously vaccinated children, we requested parents to complete symptom diaries for 7 days after vaccination. A copy of the symptom diary is presented in the Supplementary material.

## Methods

### Outbreak setting

There were 41 children (19 male, 22 female) registered at the nursery, aged 9 months to 9 years, in addition to nine staff members (all female) aged 19 to 53 years. Both cases attended the same nursery, with children grouped by age into three rooms: (i) younger than 2 years old, (ii) 2–3 years old, and (iii) 3 years and older. Additionally, some children from the local primary school attended the nursery for ‘wrap-around’ care before and after school; they did not mix with the nursery children. Case A attended the nursery on Wednesdays and Thursdays while case B attended on Mondays, Wednesdays, Thursdays and Fridays. Both cases were in the same nursery room for 2–3-year-olds but there was mixing between all children in the nursery and all nursery staff would have had contact with the cases. Detailed assessment did not identify other social networks involving the two cases outside the nursery setting.

### Case definition

The two cases fulfilled the criteria for an IMD cluster, defined as 2 or more cases of confirmed/probable IMD within 28 days in a closed setting.

### Microbiological investigations

For the two IMD cases, clinical specimens, including blood samples for culture and meningococcal PCR testing, and throat swabs for culture, were collected as part of routine clinical management of children with suspected sepsis. Culture was attempted at the local hospital laboratory and blood EDTA samples were sent to the UKHSA national Meningococcal Reference Unit (MRU), which provides a free national PCR-testing service for patients admitted to National Health Service (NHS) hospitals with suspected IMD.

Additionally, as part of the outbreak response, bacterial pharyngeal swabs were taken for all children and staff who were offered antibiotic chemoprophylaxis and vaccination. The swabs were processed at the UKHSA-commissioned laboratory at the local NHS hospital and positive isolates were submitted to the MRU for confirmation and characterisation, including serogrouping, antibiotic susceptibility testing and whole genome sequencing. Assembled draft genomes were uploaded to the *Neisseria* database at PubMLST.org (https://pubmlst.org/organisms/neisseria-spp; PubMLST IDs 150085 and 150086). The MATS and Meningococcal Antigen Surface Expression (MEASURE) assays were used to predict strain coverage for 4CMenB and MenB-fHbp vaccines, respectively [11,12].

## Results

Invasive meningococcal disease caused by serogroup B was confirmed by a positive blood PCR 4 days after symptom onset in Case A. Case B was reported to the same HPT 3 days after IMD notification of Case A. Subsequent cultures and PCR-testing were negative for *N. meningitidis*, but the diagnosis of probable IMD remained because of the classical presentation for IMD (fever, non-blanching rash) and epidemiological link with Case A.

### Public health management

The HPT collected detailed information on all exposures and contacts with Case A in the 7 days preceding illness onset, including nursery attendance, as per routine public health investigations performed for all IMD cases. Urgent chemoprophylaxis with a single dose of ciprofloxacin was arranged for household other close contacts. As per national public health guidance following a single IMD case in an educational setting, written warn-and-inform IMD advice was shared by the HPT with nursery staff and parents/guardians on the same day as notification of Case A (Figure). The HPT worked closely with the nursery, the early years team at the local authority and local NHS services to undertake risk assessment and agree the logistics for communication with nursery staff and parents/guardians. (Figure). Similar initial public health actions were undertaken for Case B as for Case A, with ciprofloxacin prophylaxis offered to household and other close contacts and warn-and-inform advice shared with nursery staff and parents/guardians (Figure).

Additionally, as soon as the second case was reported to the HPT, an Incident Management Team (IMT) was convened to manage the outbreak, as per national public health guidance (Figure). Throughout the outbreak, the IMT regularly provided written communication to the nursery staff and parents/guardians. Local general practitioners and the two local hospital trusts were informed of the cluster and the public health actions taken, along with requests to notify the HPT urgently if other children or nursery staff presented with suspected IMD.

The IMT regularly reviewed the risk assessments, agreed on necessary public health actions and oversaw their implementation. There were 41 children (including the two cases) registered at the nursery. The IMT recommended antibiotic chemoprophylaxis for all nursery staff and students to be offered as soon as possible. The IMT considered the nursery outbreak to be unusual since both cases had been 4CMenB-vaccinated and the infecting strain was confirmed as MenB in Case A. Because Case A was only PCR-confirmed, there were limited antigenic data available for the infecting strain. Antigen sequencing directly from the PCR-positive sample identified porA subtype P1.22,9,35–1 and an allele for fHbp peptide 200 (variant 2). This fHbp variant was rare among previously-sequenced invasive MenB strains, so it was not possible to conclusively predict whether the outbreak strain was covered by the 4CMenB or MenB-fHbp vaccine.

Consequently, the IMT recommended pharyngeal swabbing before giving antibiotics to the nursery staff and children in an attempt to isolate the outbreak strain which could then be tested to predict vaccine coverage. The pharyngeal swabs could also provide an insight into carriage rates and risk of transmission in this rare outbreak. The nursery manager distributed information letters to parents/guardians and nursery staff explaining the need for antibiotic chemoprophylaxis, rationale for pharyngeal swabbing and vaccination. Parents/guardians and staff were reassured that the prophylactic antibiotics would eliminate meningococcal carriage if present. All 39 children registered at the nursery (excluding the two cases) and the nine staff members consented to antibiotic chemoprophylaxis and pharyngeal swab collection, which was done 12 days after Case A was reported to the HPT.

Given that nearly all children attending the nursery had received the 4CMenB vaccine as part of the national immunisation programme, the IMT agreed that the infecting meningococcal strain was unlikely to be covered by the 4CMenB vaccine and instead, MenB-fHbp vaccination should be offered. Once testing of carriage isolates confirmed coverage by the MenB-fHbp but not the 4CMenB vaccine, these plans were actioned. The IMT acknowledged that the MenB-fHbp vaccine was not licensed for children under 10 years and had never been given to children previously vaccinated with 4CMenB. The IMT agreed that the benefits of vaccination in preventing further IMD cases far outweighed any potential side-effects. Because of the potential for increased reactogenicity after vaccination in these children, the IMT agreed to collect information on solicited and unsolicited local and systemic adverse events using daily symptom diary cards for children as well as staff receiving the MenB-fHbp vaccine. The IMT met three times - twice to plan public health actions and then around 4 weeks after the second dose of vaccine was offered to review learnings from the incident.

### Microbiological investigations

Pharyngeal swabs from five children were positive for *Neisseria* species on initial screening using Matrix-assisted laser desorption ionization–time of flight (MALDI-TOF); the isolates were referred to the MRU for confirmation and further characterisation. Of these isolates, two were confirmed as *N. meningitidis* with the same antigenic profile as the outbreak strain from Case A. The isolates were sensitive to all tested antibiotics (penicillin, rifampicin, ciprofloxacin and cefotaxime).

The two carriage isolates were genome sequenced and were both ST-269 clonal complex (cc269), with alleles for fHbp peptide 200 and Neisserial heparin-binding antigen (NHBA) peptide 255 but were devoid of the *Neisseria* adhesin A (NadA) gene. The MATS assay showed the strain was not covered for any of the three 4CMenB vaccine recombinant antigens (relative potencies: fHbp 0.002, NHBA 0.256, NadA 0.00; positive bactericidal thresholds 0.012, 0.294, 0.009, respectively). The MEASURE assay was used to predict MenB-fHbp vaccine coverage and the strain tested positive, with very high fHbp peptide expression (mean fluorescence intensity: 53,098; vs ‘covered’ threshold = 1,000). These findings confirmed the initial IMT prediction that the outbreak strain was unlikely to be covered by the 4CMenB vaccine and more likely covered by the MenB-fHbp vaccine.

### MenB-fHbp vaccination for broader protection

Letters explaining the need for MenB-fHbp vaccination, the practical arrangements and the request for completing daily symptom diaries after vaccination were sent to nursery staff and parents/guardians. A briefing session, held with the help of local paediatricians, allowed parents/guardians to ask questions before providing written consent for their children to be vaccinated, along with arrangements for assessing any children who became unwell during or after vaccination. The MenB-fHbp vaccine was offered to all children and staff who had received antibiotic chemoprophylaxis, as well as the siblings of the two cases who had also received chemoprophylaxis as household contacts of the case and became eligible for vaccination because they attended the same nursery as the case (the UK public health guidance recommends antibiotic chemoprophylaxis but not vaccination of household contacts after a single MenB IMD case).

The local pharmacy acquired MenB-fHbp vaccine directly from the vaccine manufacturer. Rather than asking staff and families to attend their GP or another healthcare setting for vaccination, the local primary care staff held two immunisation sessions at the nursery to facilitate vaccine uptake. The HPT practitioners attended the first session to answer any questions from staff or parents/guardians. The first session was held 4 weeks after the first case was reported to the HPT and the second session was held in mid-January 2024, just under 5 weeks later. Any children who had received the 4CMenB vaccine in the preceding month as part of the national childhood immunisation programme or were due to receive the 4CMenB vaccine to complete their national immunisation schedule during this period were advised to defer MenB-fHbp vaccination until at least 4 weeks after their 4CMenB dose. Mop-up appointments for those who missed either session or had to delay their first MenB-fHbp dose were held in late January 2024.

Thirty-eight children (parents of one child declined vaccination) and nine staff received two MenB-fHbp doses (Table). None of the vaccinated staff or children developed a severe adverse reaction requiring medical attention or healthcare attendance. Additionally, two staff members and the parents of 14 children returned completed symptom diaries after the first MenB-fHbp dose; post-vaccination symptoms resolved within 4 days in most children. 

**Table:** Adverse events following MenB-fHbp vaccination among nursery children and staff during an outbreak of invasive meningococcal disease, England, November 2023 (n = 16 symptom diaries completed)

Event	Children Total n = 39	Staff Total n = 9
Completed diary cards	14 (36%)	2 (22%)
No adverse events reported	3	0
Adverse event reported^a^	Total n = 11	Total n = 2
Any Pain / swelling / redness at the site of injection	7	2
Feeling hot/feverish	6	0
Tiredness	6	0
Headache	0	0
Vomiting	1	0
Diarrhoea	1	0
Muscle aches and pains	2	0
Joint aches and pains	0	0

^a^ Each person could report more than one adverse event.

Both IMD cases had a relatively mild illness and recovered without sequalae. There were no further IMD cases associated with the outbreak.

## Discussion

We report the first use of the MenB-fHbp vaccine in young children previously vaccinated with 4CMenB to control a nursery outbreak of MenB IMD caused by a strain that was not covered by the 4CMenB vaccine. The presence of fever and non-blanching rash in both cases led to a clinical suspicion of IMD. Since both cases had been fully vaccinated with 4CMenB less than 2 years previously, there was immediate concern that the infecting MenB strain may not be covered by the 4CMenB vaccine.

The only other licensed MenB vaccine is MenB-fHbp, but it is not licensed for children under 10 years, primarily because of concerns about vaccine reactogenicity and safety in infants and toddlers [9]. We did, however, find one study confirming the immunogenicity of three doses of the MenB-fHbp vaccine given on a 0-2-6 month schedule in 1–9-year-olds, with 81.4–100% of recipients achieving a pre-defined human serum bactericidal activity (hSBA) titre threshold against the four primary meningococcal test strains [9]. Among 294 children aged 2–9 years, common side-effects (≥ 1/10) included: injection site pain, swelling and redness as well as headache, diarrhoea, vomiting, muscle pain, joint pain, fever and fatigue. Toddlers aged 1 to less than 2 years also experienced drowsiness, irritability (fussiness) and loss of or decreased appetite. Fever (≥ 38 °C) was reported in 25% of 2–9-year-olds and 37% of younger children [9]. Adverse reactions following a MenB-fHbp booster in 3–5-year-olds were similar to adverse reactions during primary MenB-fHbp vaccination given to the same children 2 years earlier [9].

As most children involved in this outbreak had already received the 4CMenB vaccine, and since the 4CMenB vaccine shares similar fHbp antigens with the MenB-fHbp vaccine, there was concern that the MenB-fHbp vaccine might be very reactogenic in these children. The IMT, however, considered the benefits of protection against such a serious infection to outweigh any potential risk of higher reactogenicity in the context of an outbreak involving a vulnerable group of young children. Additionally, since the objective of vaccination was to offer early, short-term protection during an acute outbreak, a schedule of two MenB-fHbp doses given at least 4 weeks apart was recommended, rather than the licensed schedules of either two doses (with a 6-month interval) or three doses (two doses at least 1 month apart, followed by a third dose at least 4 months after the second dose) [9].

Parents and staff were asked to voluntarily complete a 7-day symptom diary card after each vaccine dose. The families of all but one child consented to vaccination and none developed severe reactions post-vaccination. Completed diary cards were returned for 43% of vaccinated children, which is similar to the 52% return rate in a recent UKHSA outbreak response involving the offer of the live modified vaccinia Ankara (MVA-BN) vaccine (Bavarian Nordic, Hellerup, Denmark) to children exposed to a person with mpox in England [13]. The rate, severity and duration of reported symptoms were consistent with published reports after primary immunisation with the 4CMenB or MenB-fHbp vaccine in children [9]. Ultimately, it is impossible to evaluate or quantify the protection offered by MenB-fHbp vaccination against subsequent cases since the absolute risk of a third case in this or any other infectious disease outbreak, although heightened, remains very low. This is especially true given the public health interventions implemented and the offer of antibiotic chemoprophylaxis to all nursery staff and students.

Since we did not obtain an isolate from either case, we collected pharyngeal swabs from all staff and children receiving antibiotic prophylaxis with the aim of isolating the outbreak strain. The isolate could then be used to assess potential coverage by the two MenB vaccines, as well as ciprofloxacin susceptibility in case alternative antibiotic chemoprophylaxis was warranted. Meningococcal carriage is highest in adolescents and young adults, and very low in young children [14], so identifying two young children as carriers rather than nursey staff was unexpected. This indicated that the outbreak strain was circulating more widely within the nursery setting and the two carriers were either pre-symptomatic (i.e. may have gone on to develop IMD) or potential sources of transmission to others who could go on to develop IMD. As suspected, further analysis identified the outbreak strain was likely preventable by the MenB-fHbp but not the 4CMenB vaccine.

We successfully used the MenB-fHbp vaccine in a nursery outbreak caused by a MenB strain not covered by 4CMenB vaccine in toddlers who had been previously immunised with 4CMenB as part of the national infant immunisation programme in England. Our findings provide real-world data to support current UK guidelines that recommend consideration of MenB-fHbp vaccination to control MenB outbreaks in populations that have previously been vaccinated with 4CMenB. Although only a small number of children were involved, we were able, for the first time, to collect solicited and unsolicited adverse events associated with MenB-fHbp vaccination in children previously vaccinated with 4CMenB. None of the children reported severe side-effects after either MenB-fHbp dose. Additionally, the nature, rates and duration of symptoms reported were consistent with known side-effects reported previously for both vaccines [9]. Larger studies are needed to confirm our findings, alongside immunogenicity studies to better understand the degree and breadth of immune responses after MenB-fHbp vaccination in children previously vaccinated with 4CMenB and, importantly, whether one or two MenB-fHbp doses are needed for additional protection. This information would be useful for managing similar future outbreaks.

The identification of two carriers in the nursery highlights the importance of early reporting of all suspected IMD cases to local public health teams, without waiting for diagnosis confirmation, to enable early institution of antibiotic chemoprophylaxis for rapid protection against further cases. Early liaison with the national reference laboratory and surveillance teams to confirm the outbreak and plan outbreak control strategies is also critical for achieving a timely and effective outbreak response.

## Conclusions

In countries and regions with established 4CMenB immunisation programmes, 4CMenB vaccination will reduce the number of MenB IMD cases, but breakthrough cases and outbreaks in vaccinated populations will more likely not be preventable by the 4CMenB vaccine. In such situations, the MenB-fHbp vaccine may be the only vaccine available for individual protection in addition to the short-term protection offered by antibiotic chemoprophylaxis. More data are needed on the immunogenicity, reactogenicity and additional protection offered by the MenB-fHbp vaccine in children previously vaccinated with 4CMenB to provide more robust evidence-based recommendations.

## Data Availability

The data are available in the article.

## Supplementary Data

115000 Supplementary Material
