# Supplementary material for: First use of Trumenba (MenB-fHbp) vaccine to control a nursery outbreak of serogroup B invasive meningococcal disease involving children previously immunised with Bexsero (4CMenB), England, November 2023
Source: Euro Surveill. 2026 Jan 22;31(3):2500431. doi: 10.2807/1560-7917.ES.2026.31.3.2500431 (PMC12848986; doi:10.2807/1560-7917.ES.2026.31.3.2500431)

This supplementary material is hosted by *Eurosurveillance* as supporting information alongside the article ‘First use of Trumenba (MenB-fHbp) vaccine to control a nursery outbreak of serogroup B invasive meningococcal disease involving children previously immunised with Bexsero (4CMenB), England, November 2023’, on behalf of the authors, who remain responsible for the accuracy and appropriateness of the content. The same standards for ethics, copyright, attributions and permissions as for the article apply. Supplements are not edited by *Eurosurveillance* and the journal is not responsible for the maintenance of any links or email addresses provided therein.

Supplementary Table S1. Timeline of events for each case, public health actions, and laboratory results following an invasive meningococcal disease outbreak in a nursery, England, November 2023 (n = 2 cases)

**Day**	**Case 1**	**Case 2**	**Public health actions**	**Lab results**
0	Onset of symptoms - Case 1.			
4	Case 1 reported to HPT.	Onset of symptoms – Case 2.	Warn & Inform letter (single case) shared.	Case 1 PCR positive at MRU, confirmed as Meningococcal group B.
5			Health Protection Team (HPT) contact nursery / school.	
7		Case 2 reported to HPT.	HPT contacted nursery for further risk assessment.	
10			1st Incident Management Team (IMT) meeting held.<br>Letter sent to parents (40 children) and staff at nursery advising antibiotic prophylaxis.	
11			Awareness raising email to Paediatricians at Northumbria and Newcastle trusts.<br>Letter to GPs about cluster of cases / antibiotic prophylaxis session.	Case 2 negative on PCR but agreed to manage as probable case due to similar clinical presentation to case 1.<br><br>Case 1 result from Meningococcal Reference Unit (MRU) as strain

				subtype P1.22,9,35-1 and harbours Factor H Binding Protein (fHbp) peptide 200 (variant 2). Unlikely to be covered by Bexero but probable to be covered by the MenB-fHbp vaccine.
12			Antibiotic prophylaxis and swabbing session carried out (39 children; 4 staff).	
13				Preliminary results from throat swabs:<br>3 positive for Neisseria on MALDI.
16				2 further throat swabs positive for Neisseria on MALDI.
19			2nd IMT Meeting:<br>Discuss results of typing / need for vaccination.<br>Agreed two dose schedule of vaccine to be offered to all children and staff in nursery.	
25				MICs have been read and both strains sensitive to ciprofloxacin (both = 0.002mg/L).
26			Local pharmacy place order for the MenB-fHbp vaccine.<br><br>Letters explaining further results and need for vaccination sent to parents (via nursery).	The Meningococcal Antigen Typing System (MATS) results from MRU:<br>2 Neisseria meningitidis isolates from throat swabs were the same

			Consent form for two doses of vaccine included with letter.	strain as the confirmed invasive case.<br><br>Both isolates found to be MATS negative for all antigens which is highly suggestive that this strain is not covered by 4CMenB.
27			Vaccine delivered to designated pharmacy.	
33			Vaccination session held.<br>Delivered by local out of hours primary care team.<br>UKHSA HPT staff attended to answer parent / staff queries.<br>Support from Early Years team at local authority.<br>All staff vaccinated.<br>5 children not vaccinated at session – variety of reasons (timing of previous vaccinations etc).<br>Arrangements made by HPT and Primary care team to schedule catch-up appointments.	
67			Second vaccination held.	

Supplementary Figure S1. Symptoms experienced by each individual after first dose of MenB-fHbp vaccine in days (n=14 children, n=2 staff)

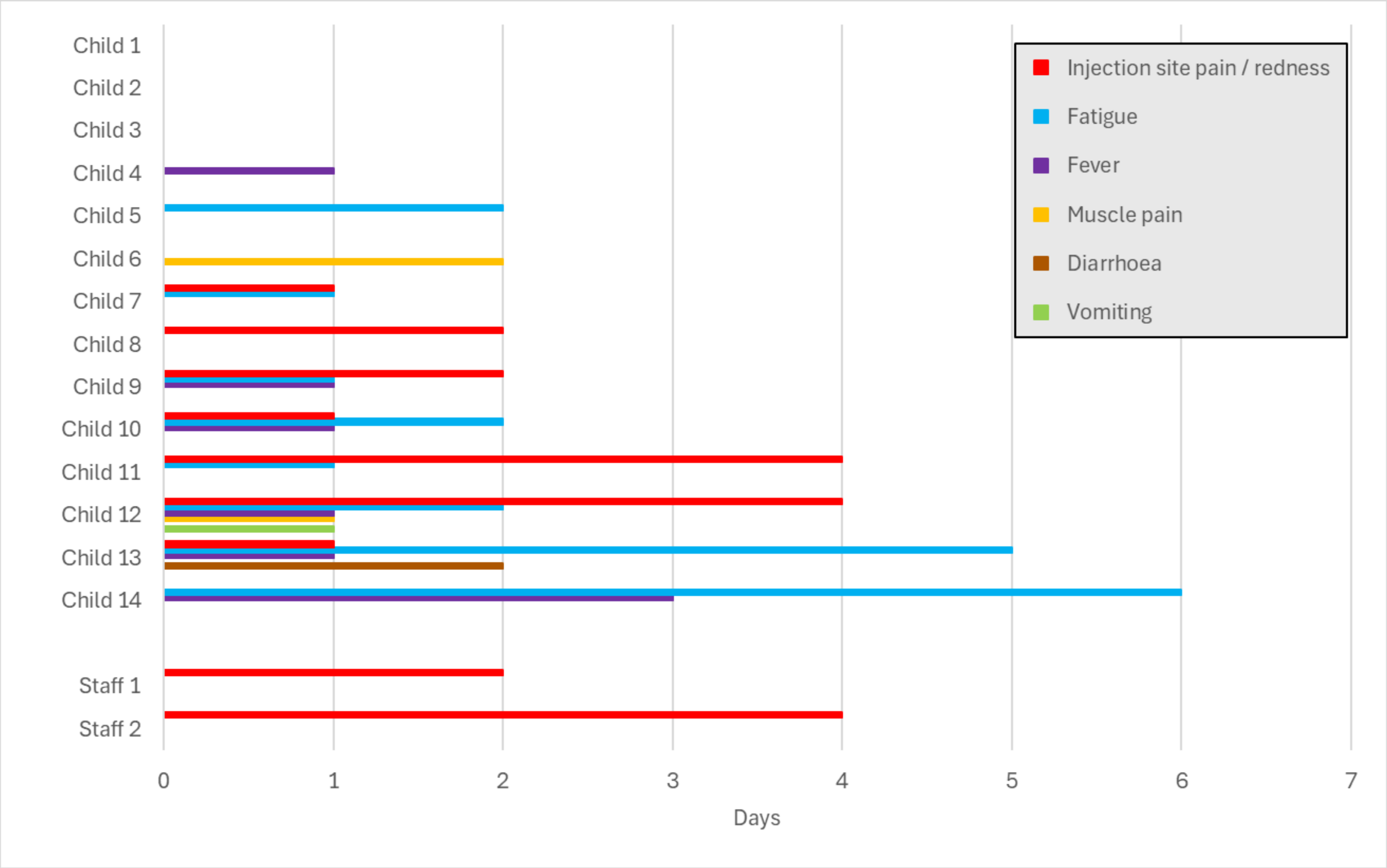

Supplement: Supplementary Material [file 25-00431_LADHANI_Supplement.pdf]
